# Pre-clinical evaluation of cyclin-dependent kinase 2 and 1 inhibition in anti-estrogen-sensitive and resistant breast cancer cells

**DOI:** 10.1038/sj.bjc.6605479

**Published:** 2009-12-15

**Authors:** N Johnson, J Bentley, L-Z Wang, D R Newell, C N Robson, G I Shapiro, N J Curtin

**Affiliations:** 1 Northern Institute for Cancer Research, Newcastle University, Paul O’ Gorman Building, Framlington Place, Newcastle upon Tyne, NE2 4HH, UK; 2 Department of Medical Oncology, Dana-Farber Cancer Institute, 44 Binney Street, Boston, MA 02115, USA

**Keywords:** breast cancer, CDK, NU2058, NU6102

## Abstract

**Background:**

Cellular proliferation, driven by cyclin-dependent kinases (CDKs) and their cyclin partners, is deregulated in cancer. Anti-estrogens, such as tamoxifen, antagonise estrogen-induced ER*α* transactivation of cyclin D1, resulting in reduced CDK4/6 activity, p27^Kip1^-mediated inhibition of CDK2 and growth arrest. We hypothesised that direct inhibition of CDK2 and CDK1 may overcome the major clinical problem of anti-estrogen resistance.

**Methods:**

The cellular effects of CDK2/1 siRNA knockdown and purine-based CDK2/1 inhibitors, NU2058 and NU6102, were measured in anti-estrogen-sensitive and resistant breast cancer cell lines.

**Results:**

CDK2 knockdown caused G1 accumulation, whereas CDK1 depletion caused G2/M slowing, and dual CDK1/2 depletion resulted in further G2/M accumulation and cell death in both anti-estrogen-sensitive and resistant cells, confirming CDK2 and CDK1 as targets for breast cancer therapy. In contrast to tamoxifen, which only affected hormone-sensitive cells, NU2058 and NU6102 reduced CDK2-mediated phosphorylation of pRb, E2F transcriptional activity and proliferation, ultimately resulting in cell death, in both anti-estrogen-sensitive and resistant cells. Both drugs caused G2/M arrest, reflective of combined CDK2/1 knockdown, with a variable degree of G1 accumulation.

**Conclusion:**

These studies confirm the therapeutic potential of CDK2 and CDK1 inhibitors for cancer therapy, and support their use as an alternative treatment for endocrine-resistant breast cancer.

Anti-estrogens, including tamoxifen, are the mainstay of treatment for hormone-dependent breast cancers. However, intrinsic and acquired anti-estrogen resistance is a significant clinical problem (Ali and Coombes, 2002). Tamoxifen competitively blocks estrogen–estrogen receptor *α* (ER*α*) binding and reduces ER*α*-mediated transcription of cell cycle genes. The mammalian cell cycle is regulated by the periodic association of cyclin-dependent kinases (CDKs) with their cyclin partners and kinase inhibitor proteins (for example, p21^Waf1/Cip1^ and p27^Kip1^). The G1/S transition is promoted by sequential CDK4/6/cyclin D1-mediated and CDK2/cyclin E-mediated phosphorylation of the retinoblastoma protein (pRb). Phosphorylation of pRb relieves transcriptional repression by the pRb–E2F complex and disrupts the binding of pRb to E2F, allowing E2F activation and transcription of genes necessary for S-phase entry and progression. In anti-estrogen-responsive breast cancers, tamoxifen causes a reduction in cyclin D1 expression, reducing CDK4/6 activity and promoting p27^Kip1^/p21^Waf1/Cip1^ inhibition of CDK2, resulting in decreased pRb phosphorylation and G1 cell cycle arrest (Planas-Silva and Weinberg, 1997).

Estrogen-independent and more aggressive breast cancers tend to have low p27^Kip1^ levels, which is an independent indicator of poor prognosis (Chiarle *et al*, 2001). Transfection of p27^Kip1^ into human mammary tumour cells caused a decrease in cyclin E/CDK2 activity, G1 accumulation and suppression of *in vivo* tumourigenicity (Carroll *et al*, 2003). Ectopic expression of p27^Kip1^ and p21^Waf1/Cip1^ may also inhibit CDK1, which has a critical role at the G2/M boundary, and has recently been implicated in G1/S control (Satyanarayana *et al*, 2008). Therefore, the direct targeting of CDK2 and CDK1 may be a useful therapeutic approach, particularly in anti-estrogen-resistant breast cancers (AERBC), where p27^Kip1^ levels are reduced.

Studies in breast cancer have highlighted CDK2 as an essential regulator of estrogen-mediated G1/S transition (Planas-Silva and Weinberg, 1997; Cariou *et al*, 2000); however, recent studies in non-breast cancer cell lines and in knockout mice have questioned the role of CDK2 in cancer cell proliferation (Berthet *et al*, 2003; Ortega *et al*, 2003; Tetsu and McCormick, 2003). To date, the effects of CDK2 knockdown in breast cancer cell lines have not been determined. Here, we examined the consequences of siRNA-mediated CDK2 knockdown in a range of anti-estrogen-sensitive and resistant breast cancer cells. Because reduced CDK2 activity can be compensated by CDK1 (Cai *et al*, 2006), we also examined the effects of CDK1 and combined CDK2/CDK1 depletion. The effects of the dual CDK2 and CDK1 inhibitors NU2058 (Arris *et al*, 2000) and NU6102 (Davies *et al*, 2002) were also compared with combined CDK2 and CDK1 knockdown. As a model of anti-estrogen resistance in the clinical setting, we used a parental MCF7 cell line and compared it with two MCF7-derived cell sublines, LCC9 and MMU2, generated by selecting for growth in the presence of anti-estrogens (Brunner *et al*, 1997; Limer *et al*, 2006). In addition, the effects of CDK1/2 knockdown and inhibition were measured in T47D (tamoxifen sensitive), MDA-MB-231 and HCC1937 (tamoxifen resistant) cell lines. Overall, the results reported in the present study provide the first evidence for the potential use of CDK2/1-specific inhibitors in AERBC.

## Materials and methods

### Materials

Radiolabelled *γ*[^32^P]ATP (specific activity=0.37 MBq *μ*l^−1^ ) was obtained from Amersham Biosciences (Buckinghamshire, UK). NU2058 and NU6102 were provided by Professor RJ Griffin (Northern Institute for Cancer Research, Newcastle University, Newcastle, UK), dissolved in DMSO at 100 mM and stored at −20°C. Tamoxifen (Sigma, Poole, UK) was dissolved in DMSO at 10 mM and stored at −20°C. Flavopiridol was provided by the Drug Synthesis and Chemistry Branch, Developmental Therapeutics Program, Division of Cancer Treatment and Diagnosis, National Cancer Institute (Bethesda, MD, USA). A 50 mmol l^−1^ stock solution in DMSO was maintained at −20°C. All other chemicals and reagents were from Sigma unless stated otherwise. Drugs were added to cells in a final DMSO concentration of 0.1%.

### Cell culture

MCF7, T47D, MDA-MB-231, HCC1937 cells, obtained from the American Tissue Culture Collection (Manassas, VA, USA) were grown in RPMI 1640 medium supplemented with 10% (v/v) fetal bovine serum, penicillin (50 U ml^−1^) and streptomycin (50 *μ*g ml^−1^) unless otherwise stated. MMU2 cells, a tamoxifen-resistant MCF7 derivative generated by selection for growth in the presence of tamoxifen (Limer *et al*, 2006), were a gift from Dr Valerie Spiers (University of Leeds, Leeds, UK). LCC9 cells, an anti-estrogen-resistant derivative of MCF7 cells selected for growth in the presence of the anti-estrogen ICI 182,780 and cross-resistant to tamoxifen (Brunner *et al*, 1997), were a gift from Robert Clarke (Georgetown University School of Medicine, Georgetown, USA). MMU2 cells were grown in phenol red-free RPMI 1640 medium and LCC9 cells in phenol red-free Dulbecco's minimum essential medium (Gibco–Invitrogen, Paisley, UK), supplemented with dextran charcoal-stripped 10% (v/v) FCS and penicillin (50 U ml^−1^) and streptomycin (50 *μ*g ml^−1^). Before the experiment, MMU2 cells underwent two passages in full RPMI 1640 medium and LCC9 cells underwent two passages in full minimum essential medium (Gibco–Invitrogen). All cells were grown in fully supplemented media during the experimental procedures.

### Growth inhibition and colony assays

To measure growth inhibition, cells were exposed to varying concentrations of tamoxifen, NU2058 and NU6102, or 0.1% (v/v) DMSO, as control, for 6 days (three cell doublings) or 7 days for HCC1937 (three cell doublings), then fixed and stained with sulforhodamine B, as described previously (Skehan *et al*, 1990). To measure cell survival after 24-h drug exposure or at 72 h after siRNA treatment, 1000 or 5000 cells were seeded in 10-cm dishes. At 2 weeks after plating, cells were fixed with 3 : 1 methanol–acetic acid and stained with 0.4% crystal violet to assess colony formation. The concentration required to inhibit cell growth by 50% (GI_50_) or reduce colony formation by 50% (LC_50_), was calculated from point-to-point graphs using GraphPad Prism (San Diego, CA, USA) software.

### Cell cycle analysis and western blotting

Cell cycle analyses and western blotting were carried out as described previously (Cai *et al*, 2006). Treatments are described in individual figure legends, and for western blotting, cells were probed with primary antibodies cyclin D1, p27^Kip1^, p21^Waf1/Cip1^ (Cell Signaling, Baltimore, MA, USA); total pRb (BD Pharmingen, San Diego, CA, USA); ppRb T821 (Biosource, Bethesda, MD, USA); ppRb S807/811 (New England Biolabs, Beverly, MA, USA); Cyclin D1, p53, ER*α* (Dako, Carpinteria, CA, USA); actin (Sigma); CDK4, CDK2, CDK1, Cyclin A, Cyclin E, total RNA polymerase II (Santa Cruz, Santa Cruz, CA, USA); RNA polymerase II [pSer^2^] (Abcam, Cambridge, MA, USA); RNA polymerase II [pSer^5^] (Covance, San Diego, CA, USA)). Blots were incubated with peroxidase-conjugated swine anti-rabbit or mouse secondary antibody (Dako). Chemiluminescence was detected using a dark box with a CCD camera (Fuji LAS 3000, Raytek, UK) and quantified using Aida image analyser software (Raytek, UK).

### E2F luciferase reporter gene assay

Cells were transfected with the E2F reporter construct (200 ng) (Hofman *et al*, 2001) together with the *β*-galactosidase construct (200 ng) (Brady *et al*, 1999) using FuGENE6 transfection reagent (Roche Diagnostics, Lewes, UK) and treated as indicated in figure legends. Luciferase activity was determined after addition of 50 *μ*l luciferase reagent (Promega, Southampton, UK) using a microplate luminometer (Perkin-Elmer, Beaconsfield, UK). To monitor the transfection efficiency, lysates were assayed for *β*-galactosidase activity by addition of *β*-galactosidase reagent and incubated at 37°C for 45 min before terminating the reaction with 1 M Na_2_CO_3_. Absorbance at 450 nm was read on a microtitre plate reader (Bio-Rad, Hemmel Hempstead, UK). Luciferase activity was normalised to the *β*-galactosidase activity and expressed as a percentage of the DMSO control.

### siRNA-mediated CDK knockdown

Cells were seeded in six-well plates and allowed to adhere for 24 h. siRNA double-stranded, annealed RNA oligonucleotides, Smart pool siRNA from Dharmacon (Chicago, USA) (CDK1, no. L-003224-00; CDK2, no. L-003236-00), were diluted in full media to a final concentration of 20 nM and mixed with RNAifect transfection reagent (Qiagen, Cambridge, UK), then added at 7 *μ*l ml^−1^ in RPMI to the cells for 12 h before replacing with fresh medium.

### CDK immunoprecipitation and kinase assays

Cyclin-dependent kinase immunoprecipitation and cdk kinase assays were carried out as described previously (Cai *et al*, 2006). X-ray films were analysed and quantified using a CCD camera (Fuji LAS 3000) and Aida image analyser software.

### Statistical analysis

Statistically significant changes were determined by unpaired Student's *t*-test using GraphPad Prism software. Statistical significance is given by ^*^P⩽0.05, ^**^P⩽0.01, ^***^P⩽0.001.

## Results

### Individual and combined CDK2 and CDK1 depletion results in cell cycle arrest in anti-estrogen-sensitive and resistant breast cancer cell lines

**Figure 1:**
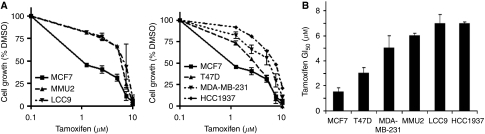
Effect of tamoxifen on breast cancer cell lines growth. (**A**) Inhibition of breast cancer cell growth by tamoxifen after 6 days. Data are mean and s.e. of three independent experiments. Left – graphs showing MCF7, MMU2 and LCC9 cell growth inhibition. Right – graphs showing MCF7, T47D, MDA-MB-231 and HCC1937 cell growth inhibition. (**B**) Tamoxifen concentrations required to inhibit breast cancer cell growth by 50% of that of vehicle-treated control over a 6-day period. Bars show mean and s.e. of concentrations from three independent experiments.

First, we confirmed the anti-estrogen sensitivity status of a panel of breast cancer cell lines. MCF7 and T47D cell lines were considered tamoxifen sensitive (GI_50_⩽3 *μ*M), whereas MMU2, LCC9, MDA-MB-231 and HCC1937 were tamoxifen resistant (GI_50_>3 *μ*M) (Figure 1).

**Figure 2:**
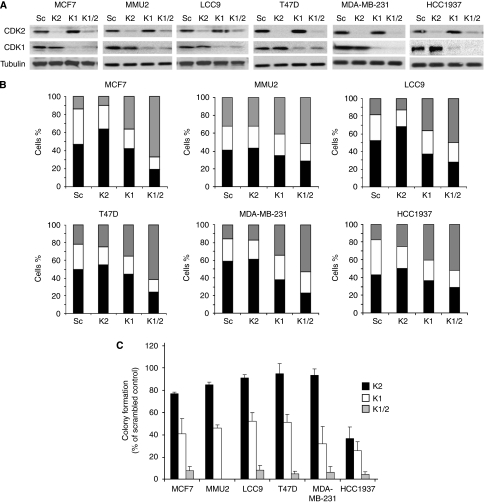
Effect of cyclin-dependent kinase-2 (CDK2), CDK1 and combined CDK2/1 siRNA on cell cycle profile and cell survival. (**A**) Representative examples of western blot showing CDK2 and CDK1 levels prepared 72 h post-transfection of either scrambled (Sc), CDK2 (K2), CDK1 (K1), CDK1 and CDK2 (K1/2) siRNA in asynchronously growing breast cancer cells. (**B**) Cell cycle analysis data pooled from three independent experiments expressed as the mean G1 (solid bar), S (open bar) and G2/M (grey bar) cell cycle fractions of cells treated as given in **A**. (**C**) Mean and s.e. of colony formation for cells treated as given in **A**; colony formation was assessed at 2 weeks post-transfection.

To evaluate the importance of CDK2 and CDK1 in breast cancer cell growth and their validity as a drug targets in breast cancer, CDK2 and CDK1 protein levels were transiently knocked down with siRNA treatment, either individually or in combination, in the exponentially growing breast cancer cell lines. All cell lines showed substantial knockdown of CDK2 and CDK1 (Figure 2A). Cyclin-dependent kinase-2 knockdown caused significant G1 accumulation in MCF7 (1.4-fold^**^) and LCC9 (1.3-fold^**^) cell lines, with only marginal increase in T47D (1.1-fold) and HCC1937 (1.2-fold) cell lines. There were corresponding significant reductions in S-phase fractions in MCF7 (1.5-fold^**^), LCC9 (1.6-fold^***^), T47D (1.4-fold^**^) and HCC1937 (1.6-fold^**^) cell lines. Cyclin-dependent kinase-2 knockdown did not affect the cell cycle pattern of MMU2 and MDA-MB-231 cell lines. Cyclin-dependent kinase-1 knockdown caused very significant G2/M accumulation in all cell lines – MCF7 (2.6-fold^***^), MMU2 (1.3-fold^**^), LCC9 (2-fold^***^), T47D (1.6-fold^**^), MDA-MB-231 (2-fold^**^) and HCC1937 (2.3-fold^***^) – with co-depletion of both CDK2 and CDK1 together, causing the greatest increases in G2/M cell cycle fractions of MCF7 (4.8-fold^***^), MMU2 (1.6-fold^***^), LCC9 (2.8-fold^***^), T47D (2.8-fold^***^), MDA-MB-231 (3.3-fold^***^) and HCC1937 (2.9-fold^***^) cell lines (Figure 2B).

Cyclin-dependent kinase-2 depletion also reduced colony formation to varying degrees in all cell lines (MCF7-23%, MMU2-15%, LCC9-9%, T47D-5% MDA-MB-231-6% and HCC1937-63% reduction in colony formation). Cyclin-dependent kinase-1 depletion further reduced colony formation to a similar degree in all cell lines (>40% reduction). Combined CDK2 and CDK1 depletion vastly diminished the number of colony-forming cells in all cell lines (>90% reduction) (Figure 2C).

### CDK1/2 inhibition reduced cell proliferation and colony formation irrespective of anti-estrogen sensitivity status

**Figure 3:**
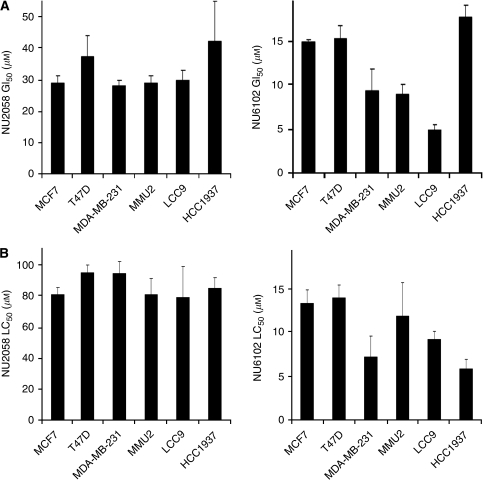
NU2058 and NU6102 concentrations required to inhibit breast cancer cell line growth and reduce cell survival by 50%. (**A**) Concentrations required to inhibit cell growth by 50% of that of vehicle-treated control over a 6-day period. Bars show mean and s.e. of concentrations from three independent experiments. Left – NU2058. Right – NU6102. (**B**) Concentrations required to reduce colony formation by 50% of that of vehicle-treated control. Cells were treated with NU2058 or NU6102 for 24 h and then replated and 2 weeks later, colony formation was assessed. Bars show mean and s.e. of concentrations from three independent experiments. Left – NU2058. Right – NU6102.

Combined CDK1/2 depletion caused cell cycle arrest and cell death in both anti-estrogen-sensitive and resistant breast cancer cell lines tested. We therefore set out to investigate whether this could be mimicked using small-molecule CDK1/2 inhibitors, NU2058 and NU6102 (Supplementary Table 1 and Supplementary Figure 1). In contrast to tamoxifen, NU2058 was almost equally potent against all breast cancer cell lines irrespective of their anti-estrogen status, with GI_50_ values ranging between 29 and 42 *μ*M. NU6102 was a more potent inhibitor of cell growth, with GI_50_ values of 5–18 *μ*M across the panel (Supplementary Figure 2A, B and Figure 3A). Furthermore, NU2058 reduced colony formation, indicative of cell death, with LC_50_ values ranging between 78 and 94 *μ*M. Similarly, NU6102 reduced cell survival, with LC_50_ values ranging between 6 and 14 *μ*M (Supplementary Figure 2C, D and Figure 3B).

**Figure 4:**
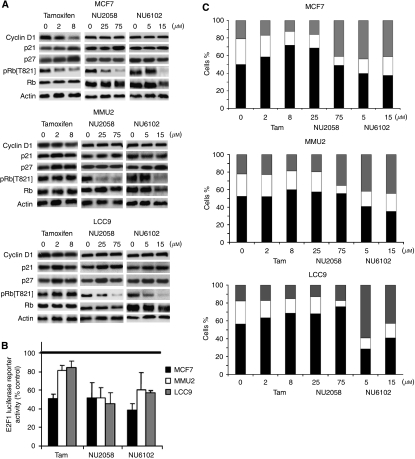
Cell cycle effects of tamoxifen, NU2058 or NU6102 on asynchronously growing MCF7 cell lines. (**A**) Representative western blot of cyclin D1, p21^Waf1/Cip1^, p27^Kip1^, ppRb T821 and total pRb protein levels in asynchronously growing MCF7, MMU2 and LCC9 cells exposed to 2 or 8 *μ*M tamoxifen, 25 or 75 *μ*M NU2058 and 5 or 15 *μ*M NU6102 for 24 h and compared with 0.1% (v/v) DMSO control. (**B**) E2F activity in cells transfected with an E2F luciferase reporter construct and a *β*-gal reporter plasmid. Relative luciferase activity was then expressed as a percentage of the DMSO-treated control. Data are mean±s.e. of three independent experiments. (**C**) Flow cytometric analyses of cells treated with tamoxifen and cyclin-dependent kinases-1/2 (CDK2/1) inhibitors expressed as the mean G1 (solid bar), S (open bar) and G2/M (grey bar) cell cycle fractions from three independent experiments.

We next went on to measure the effects of tamoxifen, NU2058 and NU6102 on cyclin D1, p21^Waf1/Cip1^, p27^Kip1^ and pRb phosphorylation at T821 – a preferential CDK2-mediated phosphorylation site (Zarkowska and Mittnacht, 1997). For these studies, we focused on the parental anti-estrogen-sensitive MCF7 cell line and its anti-estrogen-resistant derivatives, MMU2 and LCC9 cells, because (a) their common origin provided a more similar genotype/phenotype for the characterisation of CDK inhibitors and (b) their derivation mimicked the acquisition of anti-estrogen resistance clinically (Figure 4A).

Exposure of asynchronous cells for 24 h to tamoxifen at 2 *μ*M (∼GI_50_ in MCF7 cells) and 8 *μ*M (∼GI_50_ in MMU2 and LCC9 cells) (Figure 1) (Johnson *et al*, 2008) reduced cyclin D1 and increased p21^Waf1/Cip1^ and p27^Kip1^ levels; consequently, CDK2 activity was diminished, reflected by decreased pRb^pThr821^ protein levels in parental MCF7 cells. Tamoxifen had little or no effect on protein levels measured in MMU2 and LCC9 cells. There was also little or no change in cyclin D1, p21^Waf1/Cip1^ and p27^Kip1^ levels in all cell lines treated with NU2058 or NU6102. In contrast, 25 *μ*M (∼GI_50_ concentration) and 75 *μ*M NU2058 (∼GI_90_ concentration) reduced pRb^pThr821^ levels to a similar extent in all cells. Exposure to 5 *μ*M NU6102 reduced the levels of pRb^pThr821^ in the more sensitive LCC9 cell line (GI_50_∼5 *μ*M); 15 *μ*M NU6102 was required to detect reduced pRb^Thr821^ phosphorylation in MCF7 (GI_50_∼15 *μ*M) and MMU2 (GI_50_∼10 *μ*M) cells (Figure 4A). Downstream of pRb phosphorylation, E2F transcriptional activity was reduced by tamoxifen (2 *μ*M) to a greater extent in MCF7 cells than in the hormone-resistant cells, whereas NU2058 (75 *μ*M) and NU6102 (15 *μ*M) caused similar reductions in all of the cell lines (Figure 4B).

The changes in the levels of phosphorylated pRb and E2F activity were reflected in changes in cell cycle distribution (Figure 4C). In MCF7 cells, tamoxifen caused a concentration-dependent increase in G1 (2 *μ*M, 1.27-fold^*^ and 8 *μ*M, 1.57-fold^***^) and reduction in S-phase (2 *μ*M, 1.17-fold and 8 *μ*M, 1.87-fold^**^) fractions. However, in MMU2 and LCC9 cells, even at 8 *μ*M tamoxifen only caused a marginal increase in G1 accumulation (MMU2, 1.1-fold; LCC9, 1.19-fold) and S-phase depletion (MMU2 (8 *μ*M), 1.23-fold, LCC9, 1.37-fold^*^).

Treatment with 25 *μ*M NU2058 caused significant G1 accumulation in MCF7 (1.48-fold^*^) and LCC9 (1.21-fold^*^) cell lines and a corresponding decrease in S-phase fraction in MCF7 (2.2-fold^*^) and LCC9 (1.32-fold^*^) cell lines, but no significant change in MMU2 cells. Exposure to 75 *μ*M NU2058 caused a further G1 accumulation in LCC9 cell lines (1.36-fold^***^) and G2 accumulation in MCF7 (1.63-fold^*^) and MMU2 (1.6-fold^*^) cell lines. However, there was a significant S-phase depletion in all cells – MCF7 (3.44-fold^***^), MMU2 (2.66-fold^*^) and LCC9 (3.57-fold^**^).

Treatment of asynchronous populations with 5 *μ*M NU6102 arrested cells at the G2/M boundary with an increase in G2/M fraction in MCF7 (2.33-fold^*^), MMU2 (1.95-fold^**^) and LCC9 (3.4-fold^**^) cell lines compared with the DMSO control. There was little further cell cycle perturbation caused by increasing the NU6102 concentration to 15 *μ*M (Figure 4C).

### NU2058 and NU6102 reduce pRb phopshorylation and prevent G1 exit after release from serum deprivation

**Figure 5:**
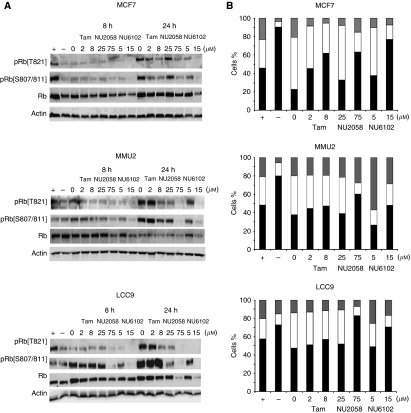
Effect of tamoxifen, NU2058 or NU6102 on phosphorylated pRb levels and cell cycle distribution in synchronously growing MCF7 cell lines. (**A**) Phosphorylated and total pRb in lysates of exponentially growing cells (+), cells serum starved for 24 h (−) or serum starved for 24 h and released into full medium in the presence of 0.1% (v/v) DMSO, 2 or 8 *μ*M tamoxifen, 25 or 75 *μ*M NU2058, 5 or 15 *μ*M NU6102 for 8 and 24 h after release. (**B**) Flow cytometric analysis of cells treated as described in **A** and harvested at 24 h after serum release, expressed as the mean G1 (solid bar), S (open bar), G2/M (grey bar) cell cycle fractions from three independent experiments.

We investigated the effects of tamoxifen, NU2058 and NU6102 on the ability of cells to transit through G1 into S-phase. We first measured pRb phosphorylation at 8 and 24 h after release from serum starvation-induced G0/1 arrest (Figure 5A). In parental MCF7 cells, at 8 h after serum addition, pRb^pSer807/811^ levels, indicative of CDK4/6 activity (Driscoll *et al*, 1999), had substantially increased, and by 24 h, pRb^pSer807/811^ and pRb^pThr821^ levels had fully recovered. Tamoxifen caused a significant decrease in pRb phosphorylation at both time points in MCF7 cells. Tamoxifen did not alter pRb phosphorylation in MMU2 or LCC9 cells at either time points. In contrast, both NU2058 and NU6102 caused concentration-dependent reductions in pRb phosphorylation after serum release in all three cell lines.

Tamoxifen significantly reduced serum-stimulated cell cycle re-entry in MCF7 cells, resulting in a significant concentration-dependent increase in the G1 population (2 *μ*M, 1.95^**^ and 8 *μ*M, 2.7-fold^**^) and reduction in S-phase fractions (2 *μ*M, 1.2^*^ and 8 *μ*M, 1.75-fold^*^) compared with the DMSO control. However, in MMU2 and LCC9 cells, tamoxifen caused only a modest retention in G1 and inhibition of progression into S-phase, which was not significant.

NU2058 had little effect at 25 *μ*M on preventing G1 exit after release from serum starvation in any of the cell lines. However, 75 *μ*M NU2058 prevented G1 exit in all cell lines, resulting in a significantly increased G1 fraction in MCF7 (2.74-fold^**^), MMU2 (1.58-fold^*^) and LCC9 (1.73-fold^**^) cell lines compared with DMSO control and reduced S-phase entry in MCF7 (2.54-fold^*^), MMU2 (3.5-fold^*^) and LCC9 (3.8-fold^*^) cell lines compared with DMSO control.

The addition of 5 *μ*M NU6102 to synchronous cultures had little effect on MCF7 cell cycle distribution. However, it caused an increase in G2/M cell cycle fractions in MMU2 (2.85-fold^*^) and LCC9 cells (1.86-fold^*^), with a corresponding decrease in the S-phase populations in MMU2 (2.65-fold^***^) and LCC9 (1.52-fold^**^) cell lines. In contrast to asynchronously growing cultures, 15 *μ*M NU6102 arrested synchronised cell populations in the G1-phase in all cells – MCF7 (3.34-fold^**^), MMU2 (1.26-fold) and LCC9 1.45-fold^*^) – compared with DMSO control and decreased the S-phase fractions in MCF7 (3.5-fold^*^), MMU2 (1.83-fold^*^) and LCC9 (2.9-fold^*^) cell lines compared with DMSO control (Figure 5B).

### NU2058 and NU6102 are equipotent CDK2 and CDK1 inhibitors in intact cells

Inhibition of human CDK2 and starfish CDK1 by NU2058 and NU6102 has previously been described (Arris *et al*, 2000; Davies *et al*, 2002; Hardcastle *et al*, 2002). Subsequent evaluation of these inhibitors against human CDK1 showed that NU6102 is 50-fold more active against CDK2/cyclin E than CDK1/cyclin B (IC_50_=5 and 250 nM), and that NU2058 has 1.5-fold selectivity for CDK2/cyclin E (IC_50_=17 *μ*M) over CDK1/cyclin B (IC_50_=26 *μ*M) (L-Z Wang, unpublished data). However, treatment of exponentially growing cells with NU2058 or NU6102 resulted in G2/M arrest, which is typical of combined CDK2 and CDK1 inhibition.

**Figure 6:**
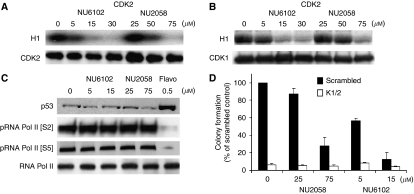
Effect of NU2058 or NU6102 on cyclin-dependent kinase-2 (CDK2) and CDK1 kinase activity, p53 and RNA polymerase II phosphorylation levels in MCF7 cells. (**A**) CDK2 and (**B**) CDK1 immunoprecipitated from control MCF7 cells or from those treated with NU6102 or NU2058 for 1 h. Immunoprecipitated CDK levels were analysed by western blotting and kinase activity was measured by CDK kinase assay. CDK protein was incubated with *γ*[^32^P]ATP and histone H1, and analysed by SDS–PAGE and autoradiography. (**C**) Western blot for p53, phospho-Serine 2 and 5 of RNA polymerase II and total RNA polymerase II protein levels measured in MCF7 cells. Lysates were collected 16 h after exposure of exponentially growing cells to NU2058 (25 and 75 *μ*M), NU6102 (5 and 15 *μ*M) and flavopiridol (0.5 *μ*M). (**D**) MCF7 cells were transfected with either scrambled or CDK1 and CDK2 siRNA; at 3 days post-transfection, cells were treated with either DMSO, NU2058 or NU6102 for 24 h followed by replating. Colony formation was assessed 2 weeks after replating. Graph shows mean and s.e. of colony formation relative to the scrambled DMSO-treated control plates.

We therefore investigated CDK2 and CDK1 inhibition by NU2058 and NU6102 in whole cells by immunoprecipitating CDK2 and CDK1 from intact MCF7 cells, which had been treated with inhibitors for 1 h, and measuring their ability to phosphorylate Histone H1. The concentrations required to inhibit CDK2 by 50% were estimated as 54 *μ*M for NU2058 and 9 *μ*M for NU6102 by quantitative densitometry (Figure 6A). Strikingly, similar concentrations were required to inhibit CDK1 by 50%, that is, 65 *μ*M for NU2058 and 11 *μ*M for NU6102 (Figure 6B). Therefore, despite their selectivity for CDK2 in enzymatic assays, in MCF7 cells, both compounds were equipotent against cellular CDK2 and CDK1. Importantly, cell growth inhibition by NU2058 and NU6102 (Figure 3) was observed at concentrations that inhibited CDK2 and CDK1 in intact cells.

To determine the effects of these compounds on the activity of the transcriptional CDKs, we measured p53 accumulation and levels of RNA polymerase II phosphorylation. Neither NU2058 (25 and 75 *μ*M) nor NU6102 (5 and 15 *μ*M) affected phospho-serine 2 or 5-RNA polymerase II or p53 levels, indicating that they were not inhibiting the activity of transcriptional CDKs in cells (Figure 6C). In contrast, flavopiridol, a potent CDK9 inhibitor, did reduce RNA polymerase II phosphorylation and increased p53 levels. Furthermore, when CDK1/2-depleted cells were treated with NU2058 and NU6102 and cell survival was measured, there was no further reduction in colony formation compared with CDK1/2 knockdown alone. This provides additional evidence that inhibition of CDK1 and CDK2 is the mechanism by which NU2058 and NU6102 kill breast cancer cells (Figure 6D).

## Discussion

The development of resistance to hormonal therapies is a major problem in the treatment of breast cancer. Because anti-estrogen therapy results in a reduction in cyclin D1 levels and p21^Waf1/Cip1^/p27^Kip1^-mediated inhibition of CDK2/1 activity, we sought to determine the ability of novel guanine-based CDK2/1 inhibitors NU2058 and NU6102 to target CDK2 and CDK1, and assess their therapeutic potential as alternatives to hormonal therapy in breast cancer. Cyclin-dependent kinase-2 and CDK1 were validated as therapeutic targets using transient siRNA knockdown. Studies in other cancer cell lines measuring cell cycle effects of individual CDK2 depletion showed little change in cell cycle profiles of asynchronous cells due to compensation by other CDK family members, including CDK1 and CDK4/6 (Cai *et al*, 2006). However, our data show that, in breast cancer cells, CDK2 depletion resulted in G1 accumulation, and a reduction in the S-phase fractions in both tamoxifen-sensitive MCF7 and T47D cells, and resistant LCC9 and HCC1937 cells, indicating that CDK2 knockdown or inhibition may not be as easily compensated in all cell types, and may have an important role in G1 progression in breast cancer cells compared with other types of cancer cells. Furthermore, CDK2 depletion resulted in partial cell death in these cell lines; in particular, CDK2 depletion caused a large reduction in colony formation in the anti-estrogen-resistant BRCA1-mutant HCC1937 cells. BRCA1-depleted breast cancer cells have previously been shown to be sensitive to CDK2 depletion or inhibition (Deans *et al*, 2006). Moreover, MCF7 cells which developed anti-estrogen resistance through loss of pRb expression, had increased cyclin A expression and CDK2 activation, and were still highly dependent on CDK2, but not on CDK4 activity for proliferation (Varma *et al*, 2007). These studies suggest that CDK2 inhibition may be of therapeutic value for several subgroups of AERBC.

Cyclin-dependent kinase-1 depletion reduced colony formation to a greater extent than did CDK2 depletion, probably because of the inability of CDK2 to fully compensate for CDK1 loss (Johnson *et al*, 2009). Combined CDK1 and CDK2 depletion caused massive reduction in colony formation in all cell lines, suggesting that small-molecule inhibition of CDK1 and CDK2 may be an effective strategy for treatment of both anti-estrogen-sensitive and resistant breast cancer populations.

After establishing that combined CDK2 and CDK1 depletion caused cell cycle arrest and cell death in both anti-estrogen-sensitive and resistant breast cancer cells, we investigated whether similar effects could be achieved by reducing CDK activity with small-molecule inhibitors. The anti-estrogen-resistant cells were 3–5 times less sensitive to tamoxifen compared with MCF7 cells and between 1.5 and 2.5 times less sensitive than T47D cells. However, there was very little difference in the sensitivity of the cells to NU2058 and NU6102 for both inhibition of cell proliferation and induction of cell death. Of note, MMU2 and LCC9 cells were more sensitive to NU6102 compared with their parental MCF7 cells. The progression to anti-estrogen resistance selects for further deregulation of cell cycle proteins, such as p27^Kip1^ or pRb (Arteaga, 2004; Varma *et al*, 2007), resulting in an increased dependency on CDK activity for survival, and potentially rendering these cells more sensitive to CDK inhibition.

Investigation of the underlying mechanisms of cell growth arrest focused on MCF7 cells and its anti-estrogen-resistant derivatives, MMU2 and LCC9 cells, revealed that, in contrast to tamoxifen, to which only MCF7 cells responded, NU2058 and NU6102 directly reduced pRb^pThr821^ phosphorylation, E2F transcriptional activity and cell growth in all the cells. Both compounds had little effect on cyclin D1, p21^Waf1/Cip1^ or p27^Kip1^ levels, indicating that the reduction in pRb phosphorylation and cell proliferation were a result of directly inhibiting CDK activity. Of note, at 15 *μ*M NU6102, total pRb protein levels were also reduced in all MCF7 cell types. This concentration resulted in cell death by colony assay; therefore, total pRb was probably reduced as a consequence of cells beginning to die. Further investigation revealed that the inhibitors did prevent S-phase re-entry after G0/G1 release, consistent with CDK2 inhibition, and that in asynchronous cells, they caused predominantly G2/M arrest, which was more indicative of dual CDK1 and CDK2 inhibition (Cai *et al*, 2006). We had previously observed that NU6102 inhibited CDK1-dependent nucleolin phosphorylation and CDK2-dependent pRb phosphorylation with equal efficiency in MCF7 cells (Davies *et al*, 2002), and other studies show that 8 *μ*M NU6102 inhibited CDK1-dependent phosphorylation in prostate cancer cells (Chen *et al*, 2006). We therefore investigated the effect of NU2058 and NU6102 on CDK1 and CDK2 kinase activity in intact cells. We showed that neither inhibitor was selective for CDK2 versus CDK1 inside cells, with both enzymes being inhibited by 50%, by about 60 *μ*M NU2058 and 10 *μ*M NU6102. Furthermore, in contrast to data from enzymatic assays, higher concentrations of NU2058, and in particular NU6102, were required to inhibit cellular CDK1 and CDK2. Similar discrepancies between the concentrations required for enzyme inhibition in cell-free and whole cell assays have been shown for a range of CDK inhibitors (Pevarello *et al*, 2005). Importantly, the concentrations of NU2058 and NU6102 required to inhibit cell proliferation by 50% were broadly similar to the concentrations required to inhibit CDK1 and CDK2 in intact cells. These compounds did not induce p53 accumulation or reduce RNA polymerase II phosphorylation at the concentrations tested, indicating that growth inhibition was not due to inhibition of transcriptional CDKs but was a result of direct inhibition of only CDK2 and CDK1. Furthermore, there was no increase in cell death when CDK1 and CDK2 depleted cells were treated with NU2058 or NU6102. These data provide strong evidence that the affects of NU2058 and NU6102 on breast cancer cell survival were mediated through inhibition of only CDK2 and CDK1. In summary, we have validated CDK2 and CDK1 as therapeutic targets in anti-estrogen-sensitive and resistant breast cancer cells by showing G1 and G2/M accumulation after CDK2, CDK1 or combined CDK knockdown, respectively. In addition, we have shown that the dual CDK1 and CDK2 inhibitors, NU2058 and NU6102, similarly induce cell cycle arrest and death. Importantly, these compounds inhibit the proliferation of breast cancer cell lines independent of their genetic backgrounds or sensitivity to anti-estrogens. These data provide the ‘proof of principle’ evidence that small-molecule CDK2/1 inhibitors could be a useful alternative treatment for breast cancer patients with endocrine-resistant disease.

## References

[bib1] Ali S, Coombes RC (2002) Endocrine-responsive breast cancer and strategies for combating resistance. Nat Rev Cancer 2: 101–112 12635173 10.1038/nrc721

[bib2] Arris CE, Boyle FT, Calvert AH, Curtin NJ, Endicott JA, Garman EF, Gibson AE, Golding BT, Grant S, Griffin RJ, Jewsbury P, Johnson LN, Lawrie AM, Newell DR, Noble ME, Sausville EA, Schultz R, Yu W (2000) Identification of novel purine and pyrimidine cyclin-dependent kinase inhibitors with distinct molecular interactions and tumor cell growth inhibition profiles. J Med Chem 43: 2797–2804 10956187 10.1021/jm990628o

[bib3] Arteaga CL (2004) Cdk inhibitor p27Kip1 and hormone dependence in breast cancer. Clin Cancer Res 10: 368S–371S 14734493 10.1158/1078-0432.ccr-031204

[bib4] Berthet C, Aleem E, Coppola V, Tessarollo L, Kaldis P (2003) Cdk2 knockout mice are viable. Curr Biol 13: 1775–1785 14561402 10.1016/j.cub.2003.09.024

[bib5] Brady ME, Ozanne DM, Gaughan L, Waite I, Cook S, Neal DE, Robson CN (1999) Tip60 is a nuclear hormone receptor coactivator. J Biol Chem 274: 17599–17604 10364196 10.1074/jbc.274.25.17599

[bib6] Brunner N, Boysen B, Jirus S, Skaar TC, Holst-Hansen C, Lippman J, Frandsen T, Spang-Thomsen M, Fuqua SA, Clarke R (1997) MCF7/LCC9: an antiestrogen-resistant MCF-7 variant in which acquired resistance to the steroidal antiestrogen ICI 182,780 confers an early cross-resistance to the nonsteroidal antiestrogen tamoxifen. Cancer Res 57: 3486–3493 9270017

[bib7] Cai D, Latham Jr VM, Zhang X, Shapiro GI (2006) Combined depletion of cell cycle and transcriptional cyclin-dependent kinase activities induces apoptosis in cancer cells. Cancer Res 18: 9270–9280 10.1158/0008-5472.CAN-06-1758 16982772

[bib8] Cariou S, Donovan JC, Flanagan WM, Milic A, Bhattacharya N, Slingerland JM (2000) Down-regulation of p21WAF1/CIP1 or p27Kip1 abrogates antiestrogen-mediated cell cycle arrest in human breast cancer cells. Proc Natl Acad Sci USA 97: 9042–9046 10908655 10.1073/pnas.160016897 PMC16818

[bib9] Carroll JS, Lynch DK, Swarbrick A, Renoir JM, Sarcevic B, Daly RJ, Musgrove EA, Sutherland RL (2003) p27(Kip1) induces quiescence and growth factor insensitivity in tamoxifen-treated breast cancer cells. Cancer Res 63: 4322–4326 12907598

[bib10] Chen S, Xu Y, Yuan X, Bubley GJ, Balk SP (2006) Androgen receptor phosphorylation and stabilization in prostate cancer by cyclin-dependent kinase 1. Proc Natl Acad Sci USA 103: 15969–15974 17043241 10.1073/pnas.0604193103 PMC1635111

[bib11] Chiarle R, Pagano M, Inghirami G (2001) The cyclin dependent kinase inhibitor p27 and its prognostic role in breast cancer. Breast Cancer Res 3: 91–94 11250752 10.1186/bcr277 PMC139437

[bib12] Davies TG, Bentley J, Arris CE, Boyle FT, Curtin NJ, Endicott JA, Gibson AE, Golding BT, Griffin RJ, Hardcastle IR, Jewsbury P, Johnson LN, Mesguiche V, Newell DR, Noble ME, Tucker JA, Wang L, Whitfield HJ (2002) Structure-based design of a potent purine-based cyclin-dependent kinase inhibitor. Nat Struct Biol 9: 745–749 12244298 10.1038/nsb842

[bib13] Deans AJ, Khanna KK, McNees CJ, Mercurio C, Heierhorst J, McArthur GA (2006) Cyclin-dependent kinase 2 functions in normal DNA repair and is a therapeutic target in BRCA1-deficient cancers. Cancer Res 66: 8219–8226 16912201 10.1158/0008-5472.CAN-05-3945

[bib14] Driscoll B, T’Ang A, Hu YH, Yan CL, Fu Y, Luo Y, Wu KJ, Wen S, Shi XH, Barsky L, Weinberg K, Murphree AL, Fung YK (1999) Discovery of a regulatory motif that controls the exposure of specific upstream cyclin-dependent kinase sites that determine both conformation and growth suppressing activity of pRb. J Biol Chem 274: 9463–9471 10092628 10.1074/jbc.274.14.9463

[bib15] Hardcastle IR, Golding BT, Griffin RJ (2002) Designing inhibitors of cyclin-dependent kinases. Annu Rev Pharmacol Toxicol 42: 325–348 11807175 10.1146/annurev.pharmtox.42.090601.125940

[bib16] Hofman K, Swinnen JV, Verhoeven G, Heyns W (2001) E2F activity is biphasically regulated by androgens in LNCaP cells. Biochem Biophys Res Commun 283: 97–101 11322773 10.1006/bbrc.2001.4738

[bib17] Johnson N, Cai D, Kennedy RD, Pathania S, Arora M, Li YC, D’Andrea AD, Parvin JD, Shapiro GI (2009) Cdk1 participates in BRCA1-dependent S phase checkpoint control in response to DNA damage. Mol Cell 35: 327–339 19683496 10.1016/j.molcel.2009.06.036 PMC3024055

[bib18] Johnson N, Speirs V, Curtin NJ, Hall AG (2008) A comparative study of genome-wide SNP, CGH microarray and protein expression analysis to explore genotypic and phenotypic mechanisms of acquired antiestrogen resistance in breast cancer. Breast Cancer Res Treat 111: 55–63 17899364 10.1007/s10549-007-9758-6

[bib19] Limer JL, Parkes AT, Speirs V (2006) Differential response to phytoestrogens in endocrine sensitive and resistant breast cancer cells *in vitro*. Int J Cancer 119: 515–521 16506217 10.1002/ijc.21863

[bib20] Ortega S, Prieto I, Odajima J, Martin A, Dubus P, Sotillo R, Barbero JL, Malumbres M, Barbacid M (2003) Cyclin-dependent kinase 2 is essential for meiosis but not for mitotic cell division in mice. Nat Genet 35: 25–31 12923533 10.1038/ng1232

[bib21] Pevarello P, Brasca MG, Orsini P, Traquandi G, Longo A, Nesi M, Orzi F, Piutti C, Sansonna P, Varasi M, Cameron A, Vulpetti A, Roletto F, Alzani R, Ciomei M, Albanese C, Pastori W, Marsiglio A, Pesenti E, Fiorentini F, Bischoff JR, Mercurio C (2005) 3-Aminopyrazole inhibitors of CDK2/cyclin A as antitumor agents. 2. Lead optimization. J Med Chem 48: 2944–2956 15828833 10.1021/jm0408870

[bib22] Planas-Silva MD, Weinberg RA (1997) Estrogen-dependent cyclin E-cdk2 activation through p21 redistribution. Mol Cell Biol 17: 4059–4069 9199341 10.1128/mcb.17.7.4059 PMC232259

[bib23] Satyanarayana A, Hilton MB, Kaldis P (2008) p21 Inhibits Cdk1 in the absence of Cdk2 to maintain the G1/S phase DNA damage checkpoint. Mol Biol Cell 19: 65–77 17942597 10.1091/mbc.E07-06-0525 PMC2174178

[bib24] Skehan P, Storeng R, Scudiero D, Monks A, McMahon J, Vistica D, Warren JT, Bokesch H, Kenney S, Boyd MR (1990) New colorimetric cytotoxicity assay for anticancer-drug screening. J Natl Cancer Inst 82: 1107–1112 2359136 10.1093/jnci/82.13.1107

[bib25] Tetsu O, McCormick F (2003) Proliferation of cancer cells despite CDK2 inhibition. Cancer Cell 3: 233–245 12676582 10.1016/s1535-6108(03)00053-9

[bib26] Varma H, Skildum AJ, Conrad SE (2007) Functional ablation of pRb activates Cdk2 and causes antiestrogen resistance in human breast cancer cells. PLoS One 2: e1256 18060053 10.1371/journal.pone.0001256 PMC2092387

[bib27] Zarkowska T, Mittnacht S (1997) Differential phosphorylation of the retinoblastoma protein by G1/S cyclin-dependent kinases. J Biol Chem 272: 12738–12746 9139732 10.1074/jbc.272.19.12738

