# Supplementary figures and images for: Pre-clinical evaluation of cyclin-dependent kinase 2 and 1 inhibition in anti-estrogen-sensitive and resistant breast cancer cells
Source: Br J Cancer. 2009 Dec 15;102(2):342–50. doi: 10.1038/sj.bjc.6605479 (PMC2816653; doi:10.1038/sj.bjc.6605479)

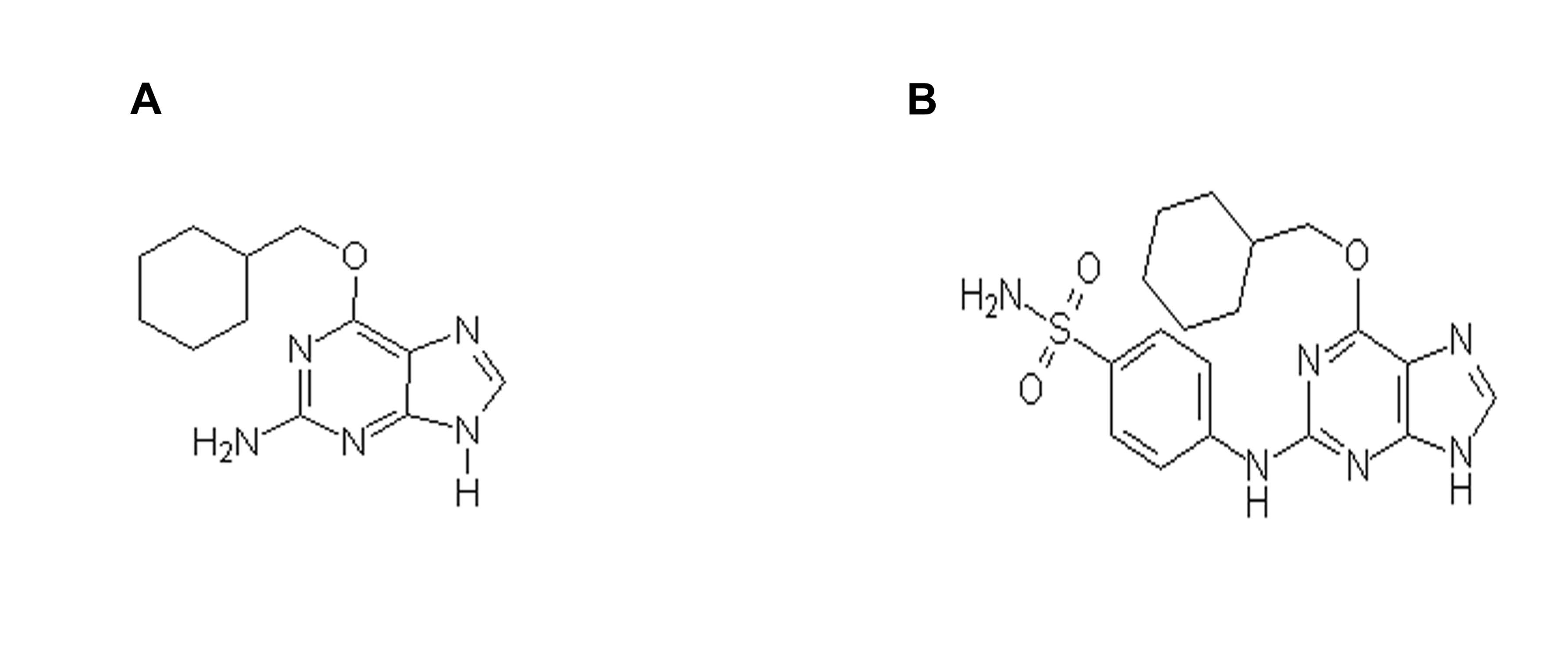

Supplement: Supplementary Figure 1 [file 6605479x1.tif]

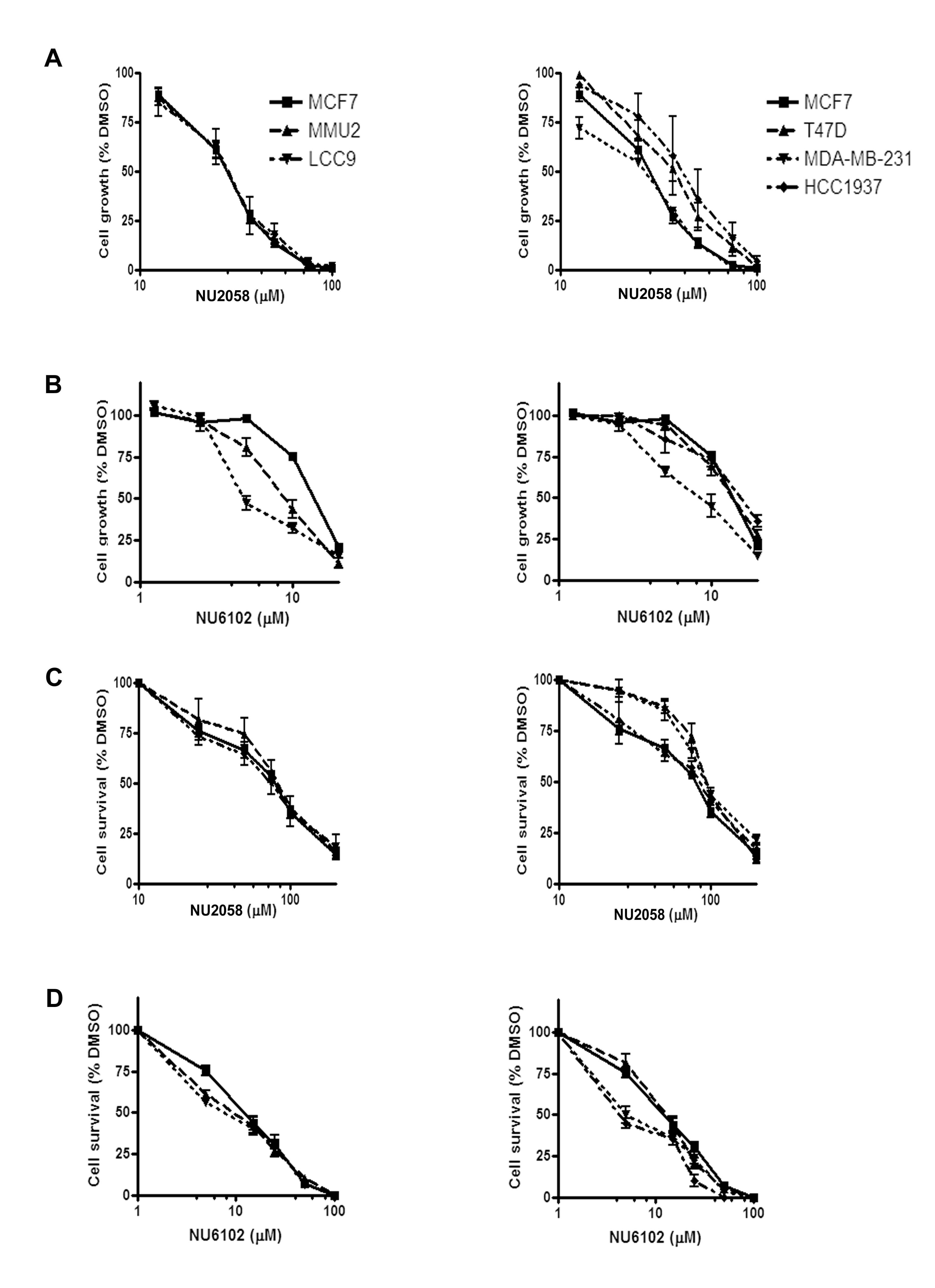

Supplement: Supplementary Figure 2 [file 6605479x2.tif]
