# Supplementary material for: Pre-clinical evaluation of cyclin-dependent kinase 2 and 1 inhibition in anti-estrogen-sensitive and resistant breast cancer cells
Source: Br J Cancer. 2009 Dec 15;102(2):342–50. doi: 10.1038/sj.bjc.6605479 (PMC2816653; doi:10.1038/sj.bjc.6605479)
Supplement: Supplementary Information [file 6605479x3.doc]

**Supplementary Figure Legends**

**Supplementary Figure 1. *Left;* NU2058 and *Right;* NU6102 chemical structure.**

**Supplementary Figure 2. Inhibition of breast cancer cell growth and survival by NU2058 and NU6102.**

A) Cells treated as for Figure 3A. Graph shows mean and S.E cell growth inhibition by NU2058 in *Left;* MCF7, MMU2 and LCC9 cells, and *Right;* MCF7, T47D, MDA-MB-231 and HCC1937 cells.

B) Cells treated as for Figure 3A. Graph shows mean and S.E cell growth inhibition by NU6102 in *Left;* MCF7, MMU2 and LCC9 cells, and *Right;* MCF7, T47D, MDA-MB-231 and HCC1937 cells.

C) Cells were treated as for Figure 3B. Graph shows mean and S.E cell survival assessed by colony formation 2 weeks post-24 hours treatment with NU2058 in *Left;* MCF7, MMU2 and LCC9 cells, and *Right;* MCF7, T47D, MDA-MB-231 and HCC1937 cells.

D) Cells were treated as for Figure 3B. Graph shows mean and S.E cell survival assessed by colony formation 2 weeks post-24 hours treatment with NU6102 in *Left;* MCF7, MMU2 and LCC9 cells, and *Right;* MCF7, T47D, MDA-MB-231 and HCC1937 cells.

**Tables**

**Supplementary Table 1. NU2058 and NU6102 *in vitro* kinase inhibition.**

| **Kinase** | **NU2058 IC 50 or % inhibition** | **NU6102 IC 50 or % inhibition** |
| --- | --- | --- |
| CDK1/cyclin B | 26 μM | 0.25 μM |
| CDK2/cyclin A3 | 17 μM | 0.005 μM |
| CDK4/cyclin D1 | 33% at 100 μM | 1.5 μM |
| CDK5/p25 | 50% at 100 μM | 0.48 μM |
| CDK7/cyclin H | 44% at 100 μM | 4.4 μM |
| CDK9/cyclin T | 43% at 100 μM | 1.1 μM |
| Kinases with IC50 values of <1 μM (IC50 value) | - | ROCK II (0.59 μM)  PDK1 (0.85 μM)  DYRK1a (0.95 μM) |
| Kinases inhibited at 10 μM (100 μM ATP):  75-100% inhibition  51-75% inhibition  25-50% inhibition | -  -  AMPK, PI3K | AMPK, CHK1, GSK3β, Lck, CSK, PI3K  JNK, MAPKAP2, MEK1, PRAK, SAPK2α, SAPK2β, SAPK4, SGK  ERK2, p70 S6K, Phos kinase, PKA, PKBα, CK1 |
